# Early care of *N*-acetyl glutamate synthase (NAGS) deficiency in three infants from an inbred family

**DOI:** 10.1016/j.ymgmr.2019.100558

**Published:** 2020-01-24

**Authors:** Katell Peoc'h, Léna Damaj, Romain Pelletier, Charles Lefèvre, Christèle Dubourg, Marie-Christine Denis, Claude Bendavid, Sylvie Odent, Caroline Moreau

**Affiliations:** a APHP, HUPNVS, UF de Biochimie Clinique, Hôpital Beaujon, F-91118 Clichy, France; b Université de Paris, U1149 INSERM, F-75018 Paris, France; c Service de Pédiatrie, Hôpital Sud, CHU Rennes Boulevard de Bulgarie, 35000 Rennes, France; d Laboratoire de Biochimie-Toxicologie, Hôpital Pontchaillou CHU Rennes, 2 rue Henri Le Guilloux, 35000 Rennes, France; e Laboratoire de Génétique moléculaire et Génomique Hôpital Pontchaillou CHU Rennes, 2 rue Henri Le Guilloux, 35000 Rennes, France; f UMR6290 IGDR, Univ Rennes, France; g Laboratoire de Biochimie Métabolique et Hormonologie, CHU, Angers, France; h Univ Rennes, INSERM, INRA, Institut NuMeCan, CHU, Rennes, France; i Service de Génétique clinique, Hôpital Sud, CHU Rennes Boulevard de Bulgarie, UMR6290 IGDR, Univ Rennes, 35000 Rennes, France

**Keywords:** *N*-acetylglutamate synthase deficiency, Urea cycle defect, Hyperammonemia, Carbaglumic acid, Prenatal diagnosis

## Abstract

*N*-acetyl glutamate synthase (NAGS) deficiency is the rarest urea cycle defect presenting as neonatal onset life-threatening hyperammonemia. We report here a family history of severe NAGS deficiency: after the index-case with severe hyperammonemia, one patient benefited from antenatal diagnosis, and from primary care at birth, another one was diagnosed at 2-days and immediately treated with carbaglumic-acid. Finally, we report excellent tolerance to long-term carbaglumic-acid treatment, with no side effects, and healthy neurological and psychomotor development.

## 1 Introduction

*N*-acetyl glutamate synthase (NAGS) deficiency is an autosomal recessive disease that usually presents as neonatal onset life-threatening hyperammonemia. NAGS catalyzes the formation of *N*-acetyl glutamate by the combination of glutamate and *N*-acetyl CoA. This molecule activates, in an allosteric manner, carbamoylphosphate synthetase I (CPSI), which is the first and rate-limiting enzyme of the urea cycle. This enzyme combines ammonia with bicarbonate to produce carbamoyl-phosphate. High plasma ammonia, glutamine, and glutamate, and barely detectable citrulline concentrations characterize NAGS deficiency [1] [2] [3] [4].

Severe NAGS deficiencies entailing the complete lack of the enzyme lead to severe neonatal hyperammonemia; late-onset presentations related to a partial lack of the enzyme have been reported in infancy, childhood, and adulthood [7]. All patients present a physiological adaptation to extra-uterine life, and neurological deterioration appears within a few hours to a few months. NAGS deficiency is the only urea cycle disorder that is curable. This disease is currently treatable using carbaglumic acid (Carbaglu®), an analog of *N*-acetyl glutamate, and a low protein diet when initiating the treatment [5]. Management of hyperammonemia is challenging in the acute phase.

We report here a family with severe NAGS deficiency within an extended inbred family. We identified three patients with a complete deficiency (homozygous for a null allele), one patient heterozygous for this variant, and numerous miscarriages and fetal deaths, probably related to the disease. This report illustrates the overall picture of the disease, its management, and the value of early prenatal or pre-symptomatic diagnosis.

## 2 Patients and methods

**Fig. 1:**
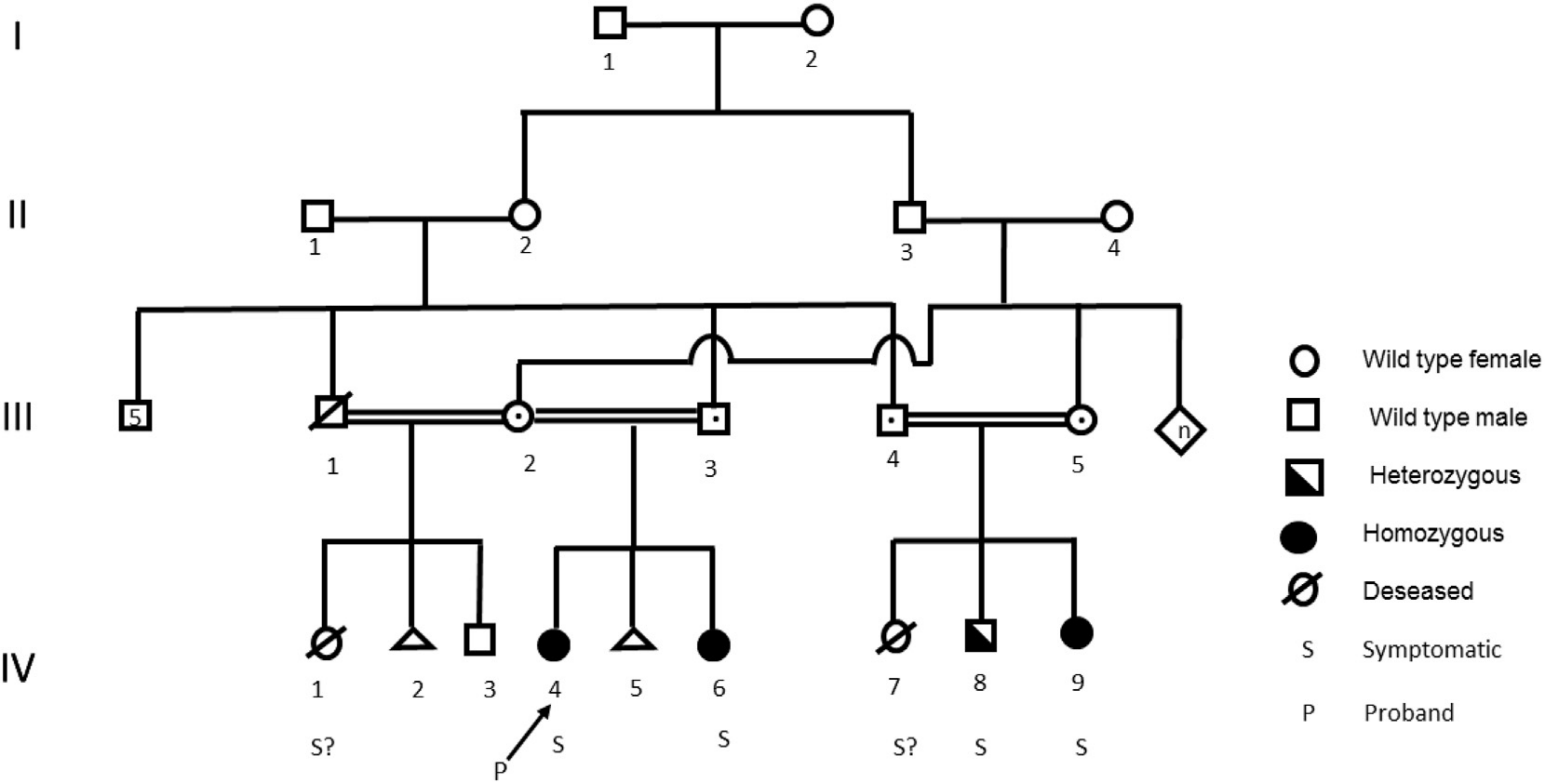
Pedigree of the family. The index case is indicated by a arrow.

We describe infants from a sizeable inbred family of Afghan descent (Fig. 1). All fathers were brothers, and the two mothers were sisters and first cousins of two of the fathers. We divided the whole family into two branches, one for each mother.

## 3 Reports and results

### 3.1 The first branch of the family

The mother of the index case reported one neonatal death in 2004 (IV-1), a miscarriage in 2005 (IV-2), and gave birth to a healthy boy (IV-3) in 2005. She then married a second brother among the siblings. She gave birth in 2013 to a baby girl (IV-4) (Fig. 1; Proband), at 39 weeks' gestation after pregnancy without any particular complications. The child's birth weight was 2770 g. At the age of 2 days, the neonate presented axial and peripheral hypotonia, weight loss of more than 10%, with alkalosis, hyperlactatemia, and major hyperammonemia (676 μmol/L; Table 1). Dialysis was immediately proposed, and she was treated with sodium phenylacetate and sodium benzoate at 250 mg/kg/day (Table1). After 24 h, ammonia fell below 200 μmol/L, treatment was adjusted (sodium benzoate 200 mg/kg/day), and Carbaglu® treatment was initiated at 100 mg/kg/day, with carnitine, arginine, and B12 vitamin supplementation. Continuous enteral nutrition without protein, with glucose and lipids, was implemented (Table1). At day-5, ammonia blood concentration normalized, and dietary proteins were progressively included in her regimen to 5 g per day. The plasma amino acid profile revealed a urea cycle disorder (Table 1), and urinary organic acid chromatography showed the absence of orotic acid. NAGS deficiency was then suspected, and the patient was found to be homozygous for the p. Thr439Hisfs*52 (NM_153006.2: c.1313dupG) *NAGS* variant, which was previously described [6]. MRI showed a discrete T2 hyper signal on palladium, and punctiform hyper signals on the periventricular white matter when the child reached the age of 7 months. She experienced a single decompensation episode and hospitalization for varicella at one year of age. Now aged six years, despite the massive hyperammonemia at birth, she has normal stature, weight, and neurological development, with an intake of 3.5 g protein/kg/day, and Carbaglu® treatment at 50 mg/kg/day.

**Table 1:** Summary of characteristics of the patients at diagnosis and during follow-up.

Patient identification	IV-4	IV-6	IV-8	IV-9
Age at first symptoms	Day 2	none	Month- 6	24 h
Clinical findings	• Axial and peripheral hypotonia • Weight loss more than 10%	Healthy baby	• Severe fatigue • Frequent crying • Unaffected by NAGS deficiency	No clinical abnormalities
Age at Sampling	Day 2	Day 1	Month- 6	Day 1
Ammonia μmol/L (11–51)	676	65	NA	117
pH (7.32–7.38)	7.55	NA	NA	7.42
Lactate mmol/L (0.5–2.2)	2.8	NA	1.3	2.1
Glutamate μmol/L (11–51)	NA	33	88	53
Glutamine μmol/L (486–670)	1907	776	854	1820
Citrulline μmol/L (10−33)	0	4	16	1
Ornithine μmol/L (47–97)	120	63	67	58
Arginine μmol/L (57–97)	40	34	45	19
*NAGS* Variant	Homozygous p.(Thr439Hisfs*52)	Homozygous p.(Thr439Hisfs*52)	Heterozygous p.(Thr439Hisfs*52)	Homozygous p.(Thr439Hisfs*52)
Acute treatment	• Dialysis • Sodium phenylacetazte and sodium benzoate (250 mg/kg/day) • Carbaglu® (100 mg/kg/day) • Carnitine (50 mg/kg/day) • Arginine (200 mg/kg/day) • B12 vitamin • nutrition without protein, with glucose (10 mg/kg/min) and lipids (2 g/kg/day)	• nutrition without protein, with glucose (10 mg/kg/min) and lipids (2 g/kg/day) • Carbaglu® (100 mg/kg/day) • Arginine (200 mg/kg/day)	none	• sodium benzoate (250 mg/kg/day) • Carbaglu® 50 mg/kg/day • Arginine (200 mg/kg/day) • Low protein diet (1 g/day)
Long-term treatment	Carbaglu® 50 mg/kg/day	Carbaglu® 50 mg/kg/day	No specific treatment or diet	Carbaglu® 50 mg/kg/day
Findings at follow-up	Normal stature, weight and neurological development, with an intake of 3.5 g protein/kg/day	Normal stature, weight and neurological development, with an intake of 3.5 g protein/kg/day	Normal stature, weight and neurological development	Normal stature, weight, and neuro-developmental evolution with no protein restriction

Then, the mother reported a miscarriage in 2014 (IV-5), and in 2015, prenatal diagnosis was performed on her fetus (IV-6), who was found to be homozygous for the p.(Thr439Hisfs*52) *NAGS* variant. Hyperechogenicity of the small intestine was found during the second-trimester ultrasound. The healthy baby girl was born by vaginal delivery (38 WA; birth weight 2915 g). Her blood ammonia concentration was monitored every 6 h for three days. The neonate was treated with a regimen with no protein, glucose, and lipids (Table1). Carbaglu® and arginine were introduced (Table1), and the mother's milk was then carefully re-introduced. At birth, she had normal ammonia levels, and amino-acid chromatography performed at day one highlighted few abnormalities presented in Table 1. She presented one decompensation episode at the age of two months (following the administration of nitrogen dioxide) with a significant seizure episode. Now four years old, she presents good stature and weight and normal neurodevelopment. She receives a normal protein intake at 3.5 g/kg/day and Carbaglu® at 50 mg/kg/day.

### 3.2 The second branch of the family

In the second branch of the family, the parents are also consanguineous (first cousins): the father was another of the siblings mentioned above, and the mother was the sister of the index case's mother. These parents reported a baby girl who died abroad on day-12 from hypotonia and coma (IV-7; 2014). Her brother (IV-8) was born in Iran in 2015 by a cesarean section (birth weight 3130 g). He presented mild hyperammonemia at six months in a context of severe fatigue and frequent crying (Table 1). Because he was heterozygous for the p.(Thr439Hisfs*52) variant of *NAGS*, we supposed his mild hyperammonemia was likely due to difficult blood sampling or delay in processing the sample. He is now aged five years and exhibits good stature, weight, and neurological development and has no specific treatment or diet.

Two years late, a baby girl was born at 39 weeks' gestation (birth weight 2844 g; IV-9) with a physiological adaptation to extra-uterine life. No prenatal diagnosis was performed because the parents initially failed to mention the family history to the physician until birth. At 24 h of life, she presented moderate hyperammonemia without alkalosis or clinical signs (Table 1). She was immediately treated with Carbaglu®, sodium benzoate, arginine and low protein diet (Table1). Before treatment, amino-acid chromatography was performed and presented abnormalities of urea cycle disorders (Table 1). The genetic diagnosis revealed homozygosity for the p.(Thr439Hisfs*52) variant of *NAGS*. She was treated with Carbaglu® (50 mg/kg/day), and arginine supplementation (58 mg/kg/day), and a low-protein regimen (2 g/kg/day) in the early stages of treatment, which was subsequently adjusted according to age and development. She presented an acute episode of hyperammonemia (153 μmol/L) at 16 months, during acute infectious disease. Now aged 20 months, she exhibits good stature, weight, and neuro-developmental evolution with no protein restriction and is treated with Carbaglu® (50 mg/kg/day).

## 4 Discussion

We present here several patients with various features of NAGS deficiency, the rarest urea cycle disorder, with an estimated incidence of 1:3,500,000–7,000,000 [3]. In our description, despite major hyperammonemia, the index case presented a favorable long-term outcome, mainly as a result of early care and treatment with N-carbamoyl glutamate. Her sister had the benefit of a prenatal diagnosis enabling management at birth to avoid hyperammonemia, without any neurological damage. To our knowledge, we report here the first prenatal molecular diagnosis that enabled successful management from birth.

The three homozygous patients reported very few decompensations with moderate hyperammonemia in hyper-catabolism situations (endogenous protein excess, fever, or intercurrent infections). Early treatment with Carbaglu® was initiated in all patients and continued over the long term to compensate for NAGS deficiency [8,9]. Finally, we report excellent tolerance for long-term treatment, with no side effects, and healthy neurological and psychomotor development. However, further studies are needed to assess the long-term outcome of these patients.
